# Exploring the feasibility of a community-based strength training program for older people with depressive symptoms and its impact on depressive symptoms

**DOI:** 10.1186/1471-2318-6-18

**Published:** 2006-11-30

**Authors:** Jane Sims, Keith Hill, Sandra Davidson, Jane Gunn, Nancy Huang

**Affiliations:** 1 School of Physiotherapy, University of Melbourne, 200 Berkeley Street, Carlton, Victoria 3053, Australia; 2 National Ageing Research Institute, PO Box 31, Parkville, Victoria 3052, Australia; 3 Department of General Practice, University of Melbourne, 200 Berkeley Street, Carlton, Victoria 3053, Australia; 4 Heart Foundation (Victoria), 411 King Street, West Melbourne, Victoria 3003, Australia

## Abstract

**Background:**

Depression is a disabling, prevalent condition. Physical activity programs may assist depression management in older people, ameliorate co-morbid conditions and reduce the need for antidepressants. The UPLIFT pilot study assessed the feasibility of older depressed people attending a community-based progressive resistance training (PRT) program. The study also aimed to determine whether PRT improves depressive status in older depressed patients.

**Methods:**

A randomised controlled trial was conducted. People aged ≥ 65 years with depressive symptoms were recruited via general practices. Following baseline assessment, subjects were randomly allocated to attend a local PRT program three times per week for 10 weeks or a brief advice control group. Follow-up assessment of depressive status, physical and psychological health, functional and quality of life status occurred post intervention and at six months.

**Results:**

Three hundred and forty six people responded to the study invitation, of whom 22% had depressive symptoms (Geriatric Depression Scale, GDS-30 score ≥ 11). Thirty two people entered the trial. There were no significant group differences on the GDS at follow-up. At six months there was a trend for the PRT intervention group to have lower GDS scores than the comparison group, but this finding did not reach significance (p = 0.08). More of the PRT group (57%) had a reduction in depressive symptoms post program, compared to 44% of the control group. It was not possible to discern which specific components of the program influenced its impact, but in post hoc analyses, improvement in depressive status appeared to be associated with the number of exercise sessions completed (r = -0.8, p < 0.01).

**Conclusion:**

The UPLIFT pilot study confirmed that older people with depression can be successfully recruited to a community based PRT program. The program can be offered by existing community-based facilities, enabling its ongoing implementation for the potential benefit of other older people.

## Background

Depressive illness results in considerable functional disability and decreased quality of life, particularly for older people. Prevalence of depression in older persons in the primary care setting ranges from 10 to 30% [1-3]. Whilst antidepressants may be effective, they can have significant side effects, such as increased risk of hip fracture [4]. Using physical activity as part of the management regimen may offset this side-effect of antidepressant medication. There is also growing evidence that physical activity can alleviate depression. For example, observational data suggest an association between physical inactivity and depression [5,6]. Several reviews have assessed the use of exercise in the management of depression and reported benefits [7-9]. The trials reviewed were of variable quality and chiefly focused on younger adults. Nevertheless, several trials indicated that physical activity can reduce depression in older people. One study focused on people who had not responded to antidepressant treatment [10]. Aerobic exercise was of short-term benefit as an adjunct treatment. In a trial of people with major depression, aerobic exercise was found to be of similar benefit to sertraline [11]. At six months follow-up, relapse rates were lower in the exercise group [12]. Secondary analyses in an osteoarthritis trial found improvements in depressive symptoms and disability at 18 months post aerobic, but not resistance, training [13]. In a brief trial with non-clinically depressed people, both walking and social contact had similar benefit [14]. An intensive strength -progressive resistance- training (PRT) program produced improvements in depression scores at 20 weeks [15]. Since polypharmacy is a management challenge amongst older people, non-pharmacological treatment options are worth considering.

None of these studies investigated whether there were additional general health and quality of life gains from the physical activity intervention for depressed older people. For older people with depression exercise may not only reduce depressive symptoms, but also improve overall health. Many health benefits are associated with physical activity in older people: lower incidence of hypertension, heart disease, osteoporosis, degenerative arthritis, colonic cancer and diabetes mellitus, improved mood and memory function, and a better maintained social network [16]. Exercise promotion is particularly pertinent in older people, where absolute risk is greatest since comorbidity is common. With increasing age, mobility, muscle power and strength decrease. There is associated decline in functionality and reduced independence, plus increased risk of injury and joint stiffness [17]. Strength training has been shown to both prevent and reverse these trends, even amongst frail older people [18]. Both aerobic and resistance training can improve functional status [19].

Whilst RCT evidence for the effectiveness of exercise in reducing depression exists, translation of this evidence into routine practice has been limited. There is a lack of evidence about the feasibility of implementing such exercise interventions in the primary health care setting. The pilot study reported here explored the ability of a community-based PRT program to reduce depression in older depressed patients. This study aimed to appraise PRT as a referral option for general practitioners (GPs) in the management of older people with depressive symptoms.

## Methods

### Participants and design

General practices across the state of Victoria, Australia were contacted by mail and telephone and invited to assist in recruiting suitable people to the study. In addition 16 Divisions of General Practice (regionally based organisations that support local GPs) assisted in informing GPs about the study, via direct contact, Divisional newsletters and Friday faxes to their members. Subject recruitment occurred via awareness raising campaigns at general practices, using information leaflets and posters; invitation letters from participating practices to *all *patients aged 65 years and over and opportunistic recruitment by GPs. Ethics approval was obtained from the University's Human Research Ethics Committee.

### Eligibility criteria

Each potential participant received a plain language statement, a consent form and a short screening instrument to assess depressive status (the 30 item Geriatric Depression Scale, GDS) [20] and suitability for exercise (Physical Activity Readiness Questionnaire, PARQ) [21]. The PARQ has 100% sensitivity and 80% specificity for the detection of contraindications to exercise [22]. On receipt of the completed screening tool, a person's eligibility was established. Inclusion criteria were: age 65 years or above and GDS score ≥ 11 [20]. We excluded all those identified as unsuitable to exercise according to the PARQ score. The participants' GPs assisted in ensuring that the following clinical exclusion criteria were met:- alcohol or drug related depression; depression with psychotic features; schizophrenia; bipolar disorder; other psychiatric diagnoses; suicidal ideation; dementia; terminally ill; uncontrolled hypertension (SBP > 210 mmHg, DBP > 110 mmHg), unstable insulin dependent diabetes (two or more hypoglycaemic episodes in the previous 3 months) and unstable angina. We excluded those currently receiving antidepressants in order to determine the independent impact of PRT.

### Baseline assessments

A researcher visited eligible participants at home and assisted their completion of the survey instruments, to provide a baseline assessment of current depressive, exercise and overall health status. To determine the intervention's impact, the instruments were mailed to all participants at 10 weeks (following the PRT program) and six months later.

### Intervention

Those allocated to the PRT intervention were referred to a local 'Living Longer, Living Stronger'™ facility. There are several hundred sites whose staff and programs are endorsed by the Council on the Ageing. The 'Living Longer, Living Stronger'™ scheme provides a framework to accredit existing community-based PRT programs to better address the needs of older people.

The study program consisted of three sessions a week for 10 weeks. A standard intervention protocol was used across all sites. The PRT entailed moderate intensity [three sets of eight/ten repetitions, at a resistance of 80% of one-repetition maximum (1-RM)] strengthening exercises using weights for the major upper and lower limb muscle groups, increased as tolerated [15]. Borg's perceived exertion scale was used to monitor intensity [23]. The program included a warm up and a warm down component. Participants paid a subsidised rate to attend (AUD $2 per session). Participants completed an attendance log and sites kept a training schedule for each person. Adherence was defined as completion of at least 60% sessions. The PRT participants received a brief telephone call each week during the 10 week program, to monitor exercise participation. They were encouraged to continue attending sessions beyond the 10 week program.

### Objectives and outcomes

The study sought to determine whether strength training improves depressive status in older people with depressive symptoms. The primary outcome was a reduction in depression. We also assessed secondary outcomes, such as improvements in physical, mental and functional health and quality of life. This implementation study design allowed us to explore the translation of effectiveness study findings into the Australian primary care setting by assessing the feasibility of older people with symptoms of depression attending a community-based strength training program.

The GDS was the study's primary outcome measure. It is an appropriate depressive symptom assessment tool for older people [20]. To obtain secondary outcome data, participants also completed the CES-D [24], the Human Activities Profile (HAP) [25], the Philadelphia Geriatric Morale Scale (PGMS) [26], the WHOQOL-BREF [27], the Self Efficacy and the Decisional Balance Scale [28] and the Physical Activity Scale for the Elderly (PASE) [29]. The HAP measures functional health status and has good discriminant validity. The PGMS assesses well-being in older people. Its subscales, agitation, attitude towards ageing and loneliness, have high internal consistency. The WHOQOL-BREF was used to measure quality of life. Its inclusion afforded comparison of our findings with international depression studies where this tool has been used. The Decisional Balance Scale, which assesses self-efficacy and motivation to exercise was included to account for mediating influences upon the main outcome measures.

### Sample size

Power calculations showed that a sample size of 130 (65 people per group) was needed to detect a 30% between group difference in depression rates (80% power, α 0.05, accounting for 30% attrition). This was based upon 40% of the control group no longer being classified as depressed at the end of the study compared with 60% of the intervention group. These calculations were based upon previous studies' findings [11-15]. Clinically significant changes on other outcome measures would be accommodated for by this sample size. Unfortunately, the funding received was only sufficient to support a small feasibility trial. This article reports on this feasibility data, plus providing some preliminary information on primary and secondary outcomes.

### Randomisation procedure and allocation concealment

All participants received ongoing care from their GP. Following baseline assessment participants were randomly allocated to receive information about exercise and local exercise options and an invitation to attend a local strength training program, or the information alone. A true 'attention control' group was not included: previous research showed the impact of PRT to be significantly greater than a program of health education sessions [15]. Randomisation was conducted centrally by an independent person in the Department of General Practice, who ascertained the person's allocation from a previously block randomised list. Participants were not blinded to their allocation and the outcome measures were based upon self-report instruments. However, those handling the outcome data were blinded to the persons' group assignment.

### Statistical analyses

Descriptive statistics were reported for all demographic and outcome variables. Intention to treat analyses was conducted. In a-priori planned analyses, linear regression model was used to examine the difference in means between the two study groups for continuous outcomes at 10 weeks and 6 months of follow-up. Given the small sample size, multivariable linear regression was used to adjust for age, gender and baseline values to protect against imbalance between these possible confounders. Differences were reported with their respective 95% Confidence Intervals (CI). In post-hoc analyses, we assessed the impact of severity and the number of PRT sessions undertaken upon the primary outcome using regression analyses (data not shown). Pearson correlation analyses were conducted to assess the association between depressive status and number of exercise sessions completed. All data analyses were conducted using SPSS™.

## Results

This was a pilot feasibility study, hence the results presented here are mainly illustrative.

### Recruitment results

**Figure 1:**
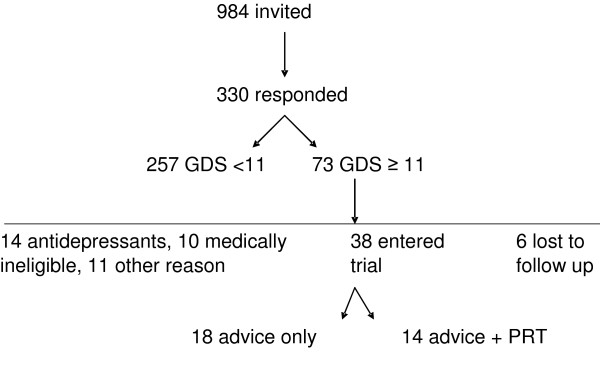
Flow chart of UPLIFT study.

General practitioners from 38 practices agreed to assist in recruitment for the study: and we recruited older people from 13 of these practices. The remainder did not find eligible people within the recruitment period due to competing demands on their time, forgetting to raise the matter with potential participants and patient refusals. Recruitment via invitation letters proved more productive than opportunistic recruitment, but not all practices had the staff capacity to assist in recruitment by this mode in accordance with the privacy/data protection legislation. Invitation letters accompanied by screening tools were sent to 984 older people. In all, 346 screening tools were returned (16 were blank) with the required consent forms. Seventy three people (22%) screened as having depressive symptoms (GDS score ≥ 11). Fourteen were already on antidepressants and 10 were medically ineligible. Eleven were unable to participate for other reasons e.g. no consent, travelling away from home, time commitments. Thirty eight people entered the trial (Figure 1). We conducted a baseline assessment with these 38 people. Four chose not to proceed to follow-up (one prior to allocation, two controls and one intervention). Two people allocated to the intervention had to delay entry and were no longer depressed at re-assessment so did not proceed. Two people were allocated to the intervention group and completed all the assessment forms but were unable to exercise. (One person acquired a health problem that prohibited her from driving, the other cited session timing constraints). Complete data, i.e. for all time points, was available for 32 people.

Ten Living Longer Living Stronger™ sites were trained to work with UPLIFT subjects: eight received clients. The remainder did not receive clients because potential participants in their area did not qualify for the study. Fourteen people were allocated to the PRT intervention (12 women) and 18 to the control group (9 women). Their mean age was 74.28 years (SD 5.87).

### Participant characteristics

There were no significant group differences on primary and secondary outcome measures at baseline. There was a greater proportion of women in the intervention group (Table 1). The average GDS score (12.41, SD 3.50) and CES-D score (17.96, SD 6.39) indicate that, in general, depressive status was of mild severity. The mean PGMS score was 9, suggesting life satisfaction and morale were slightly lower than the reported norm [26]. In terms of functional status, scores on the HAP were lower than those reported for independent living older people and somewhat closer to older people with impairment (75 and 61 respectively) [25]. The quality of life score was satisfactory for the environment domain. The score is equivalent to 69% on the full WHO-QOL. Scores were relatively low for the physical, psychological and social relationship domains. Decision balance and self-efficacy scores indicated a degree of confidence in the capacity to do physical activity under a range of circumstances. The PASE scores were somewhat higher than norms reported in the literature [29]: the intervention group had higher scores, but the difference was not significant.

**Table 1:** Baseline summary data: demographic and outcome measures

**Measure**	**Intervention Mean (SD)**	**Control Mean (SD)**	**All Mean (SD)**	**Significance (p)**
**Age (years)**	75.25 (5.78)	74.30(5.72)	74.28 (5.87)	0.63
**Gender**	Frequency	Frequency	Frequency	
**Male**	2	9	11	0.06
**Female**	12	9	21	
**GDS**†	12.64 (3.61)	12.22 (3.51)	12.41 (3.50)	0.88
**CES-D**	19.71 (6.40)	16.61 (6.22)	17.96 (6.39)	0.31
**PGMS**†*	8.77 (3.32)	9.23 (3.01)	9.03 (3.10)	0.69
**HAP MAS**	69.79 (7.64)	67.17 (9.99)	68.31(8.99)	0.12
**HAP AAS**	55.86(11.71) n = 14	51.61 (16.12) n = 18	53.47 (14.30) n = 32	0.07
WHO QOL DOM 1**	12.86 (3.27)	13.34 (2.48)	13.13 (2.81)	0.89
WHO QOL DOM 2**	13.19 (1.55)	12.86 (2.14)	13.00(1.88	0.19
WHO QOL DOM 3**	13.14 (3.08)	13.94 (3.94)	13.59 (3.55)	0.66
WHO QOL DOM 4**	14.89 (1.91)	15.62 (2.34)	15.30 (2.16)	0.46
**Self Efficacy**	16.21 (4.90)	15.88 (4.01)	16.03 (4.35)	0.35
**PASE**	145.03 (68.11)	111.25 (67.21)	126.03 (68.65)	0.06

* N = 30 (13 intervention, 17 controls)

** The four WHO-QOL domains are: physical (DOM 1), psychological (DOM 2), social (DOM 3) and environmental health (DOM 4)

† denotes a lower score is better

### Follow up

At ten weeks follow-up data were available from up to 30 people (missing data per outcome variable led to data sets for 22 to 30 people). The exploratory analyses revealed no significant differences for any of the outcome measures (Table 2). This finding persisted after adjustment (for baseline, gender and age). For both groups the PASE scores were lower than at baseline and more reflective of reported norms [29]. There was a slight improvement in functional status in both groups (p = 0.08). Reported physical activity levels decreased slightly in both groups.

**Table 2:** Primary and secondary outcome measures follow up data

	**10 weeks**			**6 month**		
	
**Measure**	**Intervention Mean (SD)**	**Control Mean (SD)**	Adjusted difference* (95% CI)	**Intervention Mean (SD)**	**Control Mean (SD)**	Adjusted difference* (95% CI)
GDS	12.23(5.22) n = 13	12.00 (4.26) n = 14	*0.59 *0.11(CI -2.59, 4.38)	11.50 (6.66) n = 14	11.88 (4.88) n = 18	*0.08 *0.35 (-0.58,8.43)
CES-D	18.27 (7.54) n = 11	15.27 (6.53) n = 15	*0.94 *0.01(CI -4.55, 4.90)	15.28 (9.90) n = 14	14.44 (7.24) n = 18	*0.55 *0.11(-4.57, 8.33)
PGMS	9.00 (3.61) n = 9	9.92 (3.15) n = 13	*0.72*-0.08 (CI -3.42, 2.42)	9.57 (4.22) n = 14	9.11 (3.66) n = 18	*0.48*-0.13 (-4.06, 1.97)
HAP MAS	72.36 (14.01) n = 14	65.47 (13.71) n = 15	*0.07*-0.28(CI -16.15, 0.70)	67.07 (9.25) n = 14	67.11(14.10) n = 18	*0.93 *0.01 (-6.83, 7.41)
HAP AAS	60.21 (19.77) n = 14	54.13 (18.45) n = 15	*0.28*-0.14(CI -14.69, 4.46)	56.64 (14.59) n = 14	54.72 (18.83) n = 18	*0.82 *0.02 (-6.15, 7.65)
WHO QOL DOM 1**	12.45 (2.00) n = 14	12.25 (1.40) n = 16	*0.36*-0.16 (CI -1.71, 0.64)	13.39 (3.03) n = 14	12.42 (2.65) n = 18	*0.05*-0.28 (-3.19, 0.01)
WHO QOL DOM 2**	12.67 (1.01) n = 14	13.04 (1.54) n = 16	*0.66 *0.09 (CI -0.81, 1.25)	13.26 (1.69) n = 14	13.37 (2.03) n = 18	*0.98*-0.003 (-1.20, 1.18)
WHO QOL DOM 3**	13.76 (3.28) n = 14	13.50 (2.10) n = 16	*0.63*-0.10 (CI -2.68, 1.65)	13.90 (3.20) n = 14	13.44 (2.04) n = 18	*0.59*-0.10 (-2.55, 1.48)
WHO QOL DOM 4**	14.97 (2.13) n = 14	14.66 (1.82) n = 16	*0.18*-0.19 (CI -1.86, 0.38)	14.39 (2.42) n = 14	14.97 (2.24) n = 18	*0.57*-0.08 (-1.80, 1.02)
Self Efficacy	14.23 (4.88) n = 13	12.36 (4.45) n = 14	*0.45*-0.16 (CI -5.43, 2.48)	13.21 (5.05) n = 14	10.81 (3.83) n = 16	*0.22*-0.26 (-6.03, 1.46)
PASE	115.76 (47.59) n = 11	97.84 (69.57) n = 15	*0.26*-0.19 (CI -63.80, 18.15)	116.39 (49.13) n = 11	104.80 (69.07) n = 18	*0.64*-0.07 (-47.86, 30.19)

* Difference between mean of intervention group and mean of control group, adjusted for gender, age and baseline value (significance in italics)

** The four WHO-QOL domains are: physical (DOM 1), psychological (DOM 2), social (DOM 3) and environmental health (DOM 4)

At six months there was a trend for the PRT intervention group to have lower GDS scores than the comparison group, but this finding did not reach significance (p = 0.08). At follow-up, 57% of the PRT group had a reduction in depressive symptoms post program, compared to 44% of the control group. The intervention group also reported better physical health than the controls.

#### Adherence to the exercise program

Adherence to the program was variable. The program entailed a maximum of 30 sessions over the 10 weeks. Five people did between 2 and 15 sessions; seven did between 18–30 sessions. Fifty eight percent met the 'adherence' criterion of 60% sessions completed. This finding compares favourably with effectiveness studies, where subjects attended a specialist facility at no cost and often with transport provided. Adherence rates are also comparable to those seen with antidepressants. No adverse events were reported. In post-hoc analysis, depressive status was negatively associated with the number of exercise sessions completed (r = -0.81, p = 0.001).

## Discussion

Our main goal was to assess the feasibility of referring older people with depressive symptoms to a PRT program. Glasgow and others have highlighted the importance of translating evidence from effectiveness studies into practice [30]. The study demonstrated that this was possible, albeit on a small scale. The study addressed several of the gaps in the literature noted by Cyarto and colleagues [31] in a recent review. In addition it provides information about the acceptance of and adherence to a structured exercise program. Effectiveness study evidence can only impact on a population's health if interventions can feasibly operate within the community.

Several process aspects will need to be addressed in a future, definitive study. These include access to exercise facilities. A few people did not have access to a car and needed to use public transport. Two people had no ready access to public transport. Community transport and taxi voucher schemes are routinely available for pension card holders in Victoria, so we made these arrangements for these people to transport them to their local exercise facility.

The adherence findings are promising: clearly more effort is involved to attend an exercise class than to take a tablet. With the exception of those who decided not to attend at all, reasons for lack of adherence were health related. They were not related to dissatisfaction with the program or lack of motivation to attend. Whilst some of these health problems may mean that people are unable to return to exercising, in many cases they were short term. There is scope for the exercise facilitators to work with these people to safely return them to an exercise program.

Whilst it was not possible to discern which specific components of the program influenced its impact, there appeared to be greater benefit amongst those adhering to the program. At 10 weeks, depressive status showed a strong negative association with the number of PRT sessions completed. An alternative explanation is that those who remained depressed participated in fewer sessions. The study's limited sample size constrains interpretation of the data, but severity of symptoms at baseline was not clearly associated with attendance. The necessary intensity or 'dose' of physical activity to improve quality of life remains unclear [32]. Several authors have reported a dose-response pattern [33,34]. Their focus has been upon differing intensities of exercise. Here, where the intensity was constant across the intervention group, the findings indicate that frequency and duration may influence depressive status.

None of the participants received any other active treatment (e.g. drugs, cognitive behaviour therapy (CBT)) for depression during the trial, so the findings reflect the impact of the PRT program. The figures for reduction in depression prevalence, whilst preliminary, are similar to those seen in trials for active treatment (e.g. drugs, CBT) and placebo respectively. Other researchers have reported on the adjunctive therapeutic benefits of exercise in those on antidepressant therapy. Since our sample only contained those not on antidepressants, further work is needed to determine whether our findings are applicable to those also on antidepressants.

We did not have sufficient power in this pilot sample to detect unequivocal group differences on the outcome measures. There is the possibility of Type II error. Caution is needed in the interpretation of the statistical findings from this exploratory study.

## Conclusion

The pilot study has demonstrated that our chosen methodology is feasible in the general practice setting, that there were some promising results in terms of the dose response of exercise and reduction in depression, and that there was a trend at six months for the intervention group to have reduced depression: these factors suggest that a larger trial is warranted. We hope to assess long term maintenance of exercise participation in a full trial and to obtain information about the influence specific elements of the program have on the primary outcome. More systematic means to encourage continuation in exercise programs are likely to be needed to optimise sustained behaviour change. The role of an enabler in assisting behaviour change is acknowledged. The positive relationships forged between clients and exercise providers suggest the potential for PRT facilitators to adopt this role. GPs can also play a role in encouraging continued attendance [35,36]. Australian GPs now have the option of using Lifescripts for prescribing physical activity to patients [37].

Other potential models to be explored in Australia include using Home and Community Care service staff, Community Access Program staff, practice nurses and other healthcare providers as enablers to maintain physical activity performance. To build on our findings, we recommend that future studies focus upon strategies to sustain adopted physical activity behaviour amongst community dwelling older people with- and without- depressive symptoms.

## Competing interests

The author(s) declare that they have no competing interest.

## Authors' contributions

JS conceived and designed the study, obtained funding and oversaw subject recruitment and analysis and prepared the paper. JG, KH, NH and SD contributed to the study design, project planning and preparation of the paper. All authors read and approved the final manuscript.

## Pre-publication history

The pre-publication history for this paper can be accessed here:


